# Supplementary material for: End Sequence Analysis Toolkit (ESAT) expands the extractable information from single-cell RNA-seq data
Source: Genome Res. 2016 Oct;26(10):1397–410. doi: 10.1101/gr.207902.116 (PMC5052061; doi:10.1101/gr.207902.116)
Supplement: Supplemental Material [file supp_gr.207902.116_Supp_Materials.tar.gz › ESAT-esat_v0.1/doc/html/classbroad_1_1core_1_1primer3_1_1_restriction_enzyme.html]

ESAT: broad.core.primer3.RestrictionEnzyme Class Reference


ESAT


- Main Page
- Related Pages
- Packages
- Classes
- Files

- Class List
- Class Index
- Class Hierarchy
- Class Members

All Classes Namespaces Files Functions Variables Enumerator Pages

- broad
- core
- primer3
- RestrictionEnzyme

Public Member Functions |
Static Public Attributes |
List of all members

broad.core.primer3.RestrictionEnzyme Class Reference

Collaboration diagram for broad.core.primer3.RestrictionEnzyme:

[legend]

|  |  |
| --- | --- |
| Public Member Functions | |
|  | RestrictionEnzyme (String name, String restrictionSite, int fivePrimeCutFromSite, int threePrimerCutFromSite) |
|  | |
| List< String > | digest (String seq) |
|  | |
| String | getName () |
|  | |

|  |  |
| --- | --- |
| Static Public Attributes | |
| static String[][] | WOBLE\_PATTERN\_MAP |
|  | |
| static RestrictionEnzyme | AcuI = new RestrictionEnzyme("AcuI", "CTGAAG", 17, 15) |
|  | |
| static RestrictionEnzyme | BpmI = new RestrictionEnzyme("BpmI", "CTGGAG", 16, 14) |
|  | |
| static RestrictionEnzyme | BpuEI = new RestrictionEnzyme("BpuEI", "CTTGAG", 16, 14) |
|  | |
| static RestrictionEnzyme | BsgI = new RestrictionEnzyme("BsgI", "GTGCAG", 16, 14) |
|  | |
| static RestrictionEnzyme | EcoP15I = new RestrictionEnzyme("EcoP15I", "CAGCAG", 27, 27) |
|  | |
| static RestrictionEnzyme | MmeI = new RestrictionEnzyme("MmeI", "TCCRAC", 20, 18) |
|  | |
| static RestrictionEnzyme | BbvI = new RestrictionEnzyme("BbvI", "GCAGC", 8, 12) |
|  | |
| static RestrictionEnzyme | BceAI = new RestrictionEnzyme("BceAI", "ACGGC", 12, 14) |
|  | |
| static RestrictionEnzyme | BsmFI = new RestrictionEnzyme("BsmFI", "GGGAC", 10, 14) |
|  | |
| static RestrictionEnzyme | BtgZI = new RestrictionEnzyme("BtgZI", "GCGATG", 10, 14) |
|  | |
| static RestrictionEnzyme | EciI = new RestrictionEnzyme("EciI", "GGCGGA", 11, 9) |
|  | |
| static RestrictionEnzyme | FokI = new RestrictionEnzyme("FokI", "GGATG", 9, 13) |
|  | |

## Constructor & Destructor Documentation

|  |  |  |  |
| --- | --- | --- | --- |
| broad.core.primer3.RestrictionEnzyme.RestrictionEnzyme | ( | String | *name*, |
|  |  | String | *restrictionSite*, |
|  |  | int | *fivePrimeCutFromSite*, |
|  |  | int | *threePrimerCutFromSite* |
|  | ) |  |  |

## Member Function Documentation

|  |  |  |  |  |  |
| --- | --- | --- | --- | --- | --- |
| List<String> broad.core.primer3.RestrictionEnzyme.digest | ( | String | *seq* | ) |  |

|  |  |  |  |  |
| --- | --- | --- | --- | --- |
| String broad.core.primer3.RestrictionEnzyme.getName | ( |  | ) |  |

## Member Data Documentation

RestrictionEnzyme broad.core.primer3.RestrictionEnzyme.AcuI = new RestrictionEnzyme("AcuI", "CTGAAG", 17, 15)

static

RestrictionEnzyme broad.core.primer3.RestrictionEnzyme.BbvI = new RestrictionEnzyme("BbvI", "GCAGC", 8, 12)

static

RestrictionEnzyme broad.core.primer3.RestrictionEnzyme.BceAI = new RestrictionEnzyme("BceAI", "ACGGC", 12, 14)

static

RestrictionEnzyme broad.core.primer3.RestrictionEnzyme.BpmI = new RestrictionEnzyme("BpmI", "CTGGAG", 16, 14)

static

RestrictionEnzyme broad.core.primer3.RestrictionEnzyme.BpuEI = new RestrictionEnzyme("BpuEI", "CTTGAG", 16, 14)

static

RestrictionEnzyme broad.core.primer3.RestrictionEnzyme.BsgI = new RestrictionEnzyme("BsgI", "GTGCAG", 16, 14)

static

RestrictionEnzyme broad.core.primer3.RestrictionEnzyme.BsmFI = new RestrictionEnzyme("BsmFI", "GGGAC", 10, 14)

static

RestrictionEnzyme broad.core.primer3.RestrictionEnzyme.BtgZI = new RestrictionEnzyme("BtgZI", "GCGATG", 10, 14)

static

RestrictionEnzyme broad.core.primer3.RestrictionEnzyme.EciI = new RestrictionEnzyme("EciI", "GGCGGA", 11, 9)

static

RestrictionEnzyme broad.core.primer3.RestrictionEnzyme.EcoP15I = new RestrictionEnzyme("EcoP15I", "CAGCAG", 27, 27)

static

RestrictionEnzyme broad.core.primer3.RestrictionEnzyme.FokI = new RestrictionEnzyme("FokI", "GGATG", 9, 13)

static

RestrictionEnzyme broad.core.primer3.RestrictionEnzyme.MmeI = new RestrictionEnzyme("MmeI", "TCCRAC", 20, 18)

static

String [][] broad.core.primer3.RestrictionEnzyme.WOBLE\_PATTERN\_MAP

static

**Initial value:**

= {

{"M", "[AC]"},

{"R", "[AG]"},

{"S", "[CG]"},

{"V", "[AGC]"},

{"W", "[AT]"},

{"Y", "[CT]"},

{"H", "[ACT]"},

{"K" , "[GT]"},

{"D" , "[AGT]"},

{"B" , "[TGC]"},

{"N" , "[ATGC]"}

}

---

The documentation for this class was generated from the following file:

- java/broad/core/primer3/RestrictionEnzyme.java


---

Generated on Fri Aug 15 2014 15:10:55 for ESAT by  

 1.8.7
